# Genomic stability at the coding regions of the multidrug transporter gene *ABCB1*: insights into the development of alternative drug resistance mechanisms in human leukemia cells

**DOI:** 10.20517/cdr.2020.51

**Published:** 2020-11-03

**Authors:** Kevin G. Chen, George E. Duran, Mark J. Mogul, Yan C. Wang, Kevin L. Ross, Jean-Pierre Jaffrézou, Lyn M. Huff, Kory R. Johnson, Tito Fojo, Norman J. Lacayo, Branimir I. Sikic

**Affiliations:** ^1^Division of Oncology, Department of Medicine, Stanford University School of Medicine, Stanford, CA 94305, USA.; ^2^Current Address: NIH Stem Cell Unit, National Institute of Neurological Disorders and Stroke, National Institutes of Health, Bethesda, MD 20892, USA.; ^3^Current Address: Medical Affairs U.S., Servier Pharmaceuticals, Boston, MA 02210, USA.; ^4^Current Address: Ross BioPharm Group, Rocky Point, NY 11778, USA.; ^5^Current Address: French National Centre for Scientific Research, Paris 75016, France.; ^6^Medicine Branch, National Cancer Institute, National Institutes of Health, Bethesda, MD 20892, USA.; ^7^Current Address, Laboratory of Cell Biology, National Cancer Institute, National Institutes of Health, Bethesda, MD 20892, USA.; ^8^Intramural IT and Bioinformatics Program, National Institute of Neurological Disorders and Stroke, National Institutes of Health, Bethesda, MD 20892, USA.; ^9^Current Address: Herbert Irving Comprehensive Cancer Center, Columbia University Medical Center/New York-Presbyterian Hospital, New York, NY 10032, USA.; ^10^Division of Pediatric Hematology-Oncology-Stem Cell Transplantation and Cancer Biology, Stanford University School of Medicine and Stanford Cancer Institute, Palo Alto, CA 94305, USA.

**Keywords:** Cancer, leukemia, multidrug resistance, *ABCB1*, P-glycoprotein, cyclosporine, mutation

## Abstract

**Aim**: Despite considerable efforts to reverse clinical multidrug resistance (MDR), targeting the predominant multidrug transporter ABCB1/P-glycoprotein (P-gp) using small molecule inhibitors has been unsuccessful, possibly due to the emergence of alternative drug resistance mechanisms. However, the non-specific P-gp inhibitor cyclosporine (CsA) showed significant clinical benefits in patients with acute myeloid leukemia (AML), which likely represents the only proof-of-principle clinical trial using several generations of MDR inhibitors. Nevertheless, the mutational mechanisms that may underlie unsuccessful MDR modulation by CsA are not elucidated because of the absence of CsA-relevant cellular models. In this study, our aims were to establish CsA-resistant leukemia models and to examine the presence or absence of *ABCB1* exonic mutations in these models as well as in diverse types of human cancer samples including AMLs.

**Methods**: Drug-resistant lines were established by stepwise drug co-selection and characterized by drug sensitivity assay, rhodamine-123 accumulation, [^3^H]-labeled drug export, *ABCB1* cDNA sequencing, and RNase protection assay. The genomic stability of the *ABCB1* coding regions was evaluated by exome sequencing analysis of variant allele frequencies in human populations. Moreover, the mutational spectrum of *ABCB1* was further assessed in diverse types of cancer samples including AMLs in the Cancer Genome Atlas (TCGA) at the National Cancer Institute.

**Results**: We report the development of two erythroleukemia variants, RVC and RDC, which were derived by stepwise co-selection of K562/R7 drug-resistant leukemia cells with the etoposide-CsA and doxorubicin-CsA drug combinations, respectively. Interestingly, both RVC and RDC cell lines, which retained P-gp expression, showed altered multidrug-resistant phenotypes that were resistant to CsA modulation. Strikingly, no mutations were found in the *ABCB1* coding regions in these variant cells even under long-term stringent drug selection. Genomically, *ABCB1* displayed relatively low variant allele frequencies in human populations when compared with several ABC superfamily members. Moreover, *ABCB1* also exhibited a very low mutational frequency in AMLs compared with all types of human cancer. In addition, we found that CsA played a role in undermining the selection of highly drug-resistant cells via induction of low-level and unstable drug resistance.

**Conclusion**: Our data indicate that *ABCB1* coding regions are genomically stable and relatively resistant to drug-induced mutations. Non-*ABCB1* mutational mechanisms are responsible for the drug-resistant phenotypes in both RVC and RDC cell lines, which are also prevalent in clinical AML patients. Accordingly, we propose several relevant models that account for the development of alternative drug resistance mechanisms in the absence of *ABCB1* mutations.

## Introduction

Multidrug resistance (MDR), a phenotype that has been well-defined both *in vitro* in cell culture and *in vivo* in cancer patients, is principally caused by numerous ATP-binding cassette (ABC) transporters^[1]^. The best characterized ABC transporter is ABCB1, known as P-glycoprotein (P-gp), which has been implicated in clinical MDR^[1-5]^. A wide range of clinical trials were conducted over the past 30 years in patients with different types of cancer by co-administration of small molecule inhibitors of P-gp and anticancer drugs. There were three generations of P-gp inhibitors that were used in these trials, including cyclosporine (CsA)^[6-11]^, the cyclosporine D analogue PSC 833 (PSC-833)^[12-15]^, and zosuquidar^[16-18]^.

Encouragingly, a significant clinical benefit was achieved in elderly refractory AML patients in a trial using the non-specific P-gp inhibitor CsA^[9]^. However, this clinical benefit was overshadowed due to the results of subsequent CsA trials under different conditions^[11]^, perhaps resulting from a flawed trial design^[19]^ and the selection of inappropriate AML subtypes. Both potential factors made CsA data interpretation quite difficult, producing inconclusive results. Moreover, despite substantial clinical efforts, other advanced phase III trials showed no favorable outcomes using highly specific P-gp inhibitors (e.g., PSC-833 and zosuquidar). Thus, it was postulated that there might be redundant drug resistance mechanisms that are responsible for resistance to MDR modulation in cancer cells^[1-5]^.

The mechanisms that underlie resistance to MDR modulation are complicated and multifactorial in nature. These underlying mechanisms comprise: (1) the genetic heterogeneity of the *ABCB1* gene (e.g., various single nucleotide polymorphisms and mutations)^[20-25]^; (2) genomic and epigenomic instability events that regulate *ABCB1* expression^[26-30]^; (3) the involvement of non-P-gp ABC transporters (e.g., *ABCA3*, *ABCC1/MRP1* and *ABCG2/BCRP*)^[31-37]^; and (4) various non-MDR modifiers that involve cell cycle control, anti-apoptosis and cytoskeleton alterations^[3,38,39]^.

Thus far, CsA-mediated modulation of MDR still represents the only proof-of-principle clinical trial of MDR modulation, but the mechanism is poorly understood, largely owing to the absence of drug-resistant models related to CsA-induced drug resistance mechanisms. Several existing MDR modulation models were based on the use of the potent and specific P-gp inhibitor PSC-833^[21,40-43]^, which provided insights into alternative drug resistance mechanisms that are not relevant to CsA modulation.

In this study, we report the establishment of two CsA-relevant models, termed RVC and RDC, which are resistant to CsA modulation. RVC was designated according to selection of K562/R7 P-gp-positive leukemia cells with the VP-16 (etoposide)-CsA combination. Similarly, RDC was named based on selection of K562/R7 cells with the doxorubicin-CsA pair. Because gene mutations play a significant role in conferring drug resistance, our original aim was to examine the presence or absence of *ABCB1* exonic mutations in these drug-resistant leukemia models. After initial characterization of their drug-resistant phenotypes, we directly sequenced *ABCB1* mRNAs in these cell lines. Strikingly, no single *ABCB1* exonic mutation was found in various stages of these drug-resistant cells, suggesting that the *ABCB1* coding regions are genomically stable and relatively resistant to drug-induced mutations.

To facilitate the understanding of the intractability of the *ABCB1* coding regions to drug-induced mutations in these leukemia cells, we compared the exonic variant allele frequencies, an indicator of genomic stability, between *ABCB1* and several representative transporter genes in human populations. We also examined the mutational spectrum of *ABCB1* in diverse types of cancer samples including AMLs in the Cancer Genome Atlas (TCGA) at the National Cancer Institute. Collectively, our findings indicate that non-*ABCB1* mutational mechanisms, responsible for the drug-resistant phenotypes in both RVC and RDC, are also prevalent in clinical AML patients. Lastly, we propose several relevant models that account for the development of alternative drug resistance mechanisms in the absence of *ABCB1* mutations.

## Methods

### Chemical reagents and drugs

The sources of anticancer drugs and chemicals were as follows: VP-16 was obtained from Bristol-Myers (Evansville, IN), doxorubicin from Adria Laboratories (Columbus, OH), vinblastine from Eli Lilly and Co. (Indianapolis, IN), PSC-833 (known as valspodar) from Sandoz Pharmaceutical Corporation (Basel, Switzerland), CsA from Sigma Chemical Co. (St. Louis, MO). and rhodamine 123 (Rh-123) from Molecular Probes (Eugene, OR). All radiolabeled reagents, namely [^3^H]-daunorubicin, [^3^H]-vinblastine and [^3^H]-etoposide, were purchased from Amersham (Arlington Heights, IL). All other chemicals were from Sigma-Aldrich (St. Louis, MO).

### Cell culture and development of drug-resistant cellular models

**Figure 1:**
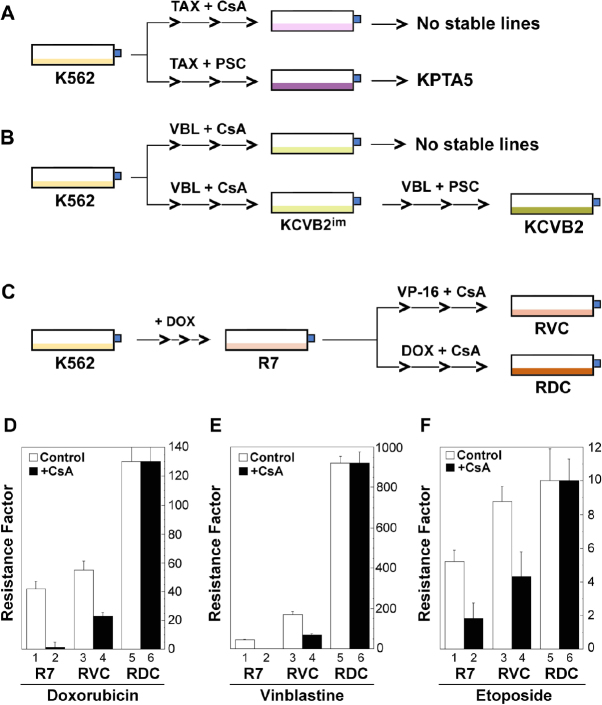
Development of drug-resistant leukemic cell lines that are insensitive to MDR modulation. A and B: development of the paclitaxel (Taxol)- and vinblastine-resistant leukemic cell lines (KPTA5 and KCVB2) in the presence of cyclosporine (CsA) (in this study) or PSC-833 (PSC)^[41,42]^; C: development of drug-resistant leukemic cell lines (RVC and RDC) by stepwise co-selection of the multidrug-resistant line K562/R7 (R7) with etoposide (VP-16) or doxorubicin (DOX) in the presence of 2 µmol/L cyclosporine (CsA); D-F: Drug resistance factors (R_f_) represent fold changes of drug resistance relative to the parental K562 cells as determined by the MTT assays. The mean values (resistance factors) and standard deviations (bars) are shown from one of the two similar experiments. Of note, vinblastine resistance in R7 with CsA (E, columns 1 and 2) was restored to parental K562 cell level, which is invisible in the 0-1000 scale

We previously developed and characterized a panel of drug-resistant cell lines from human uterine sarcoma, MES-SA and MES-SA/Dx5^[44,45]^, some of which were used as controls in this study [Table 1]. In this study, we characterized multiple drug-resistant models from the K562/R7 (R7) cell line by stepwise co-selection of R7 cells with both doxorubicin and CsA, or VP-16 (etoposide) and CsA [Figure 1]. These cells were cultured in suspension in McCoy’s medium supplemented with in 10% newborn calf serum (Life Technologies, Inc.), 2 mmol/L L-glutamine, and antibiotics (streptomycin and penicillin). Cells were maintained at 37 °C in an incubator with a humidified atmosphere and 5% CO_2_. All cultures were routinely tested for mycoplasma infection.

**Table 1:** Development of multidrug resistant cell lines that are insensitive to cyclosporine modulation

Cell lines	Drug Selection^a^	Drug Resistance^b^			ABCB1 cDNA^c^	Drug resistance mechanisms^d^
		to DOX	to VBL	to VP-16		
MES-SA*	None	1	1	1	Negative	Sensitive control
Dx5^[20]^	DOX	80	243	42	WT	MDR
DxP^[20]^	DOX, PSC	77	24	18	Mutant ^e^	Altered MDR
K562*	None	1	1	1	Negative	Sensitive control
KCVB2^i^	VBL, CsA	Unstable, NA	Unstable, NA	Unstable, NA	WT	MDR, Non-MDR
KCVB2^[41]^	VBL, PSC	8	8	1	Negative	Non-MDR
KPTA5^i^	TAX, CsA	Unstable, NA	Unstable, NA	Unstable, NA	WT	MDR, Non-MDR
KPTA5^[42]^	TAX, PSC	9	1.5	1.3	Negative	Non-MDR
K562/R7*^[42]^	DOX	1	1	1	WT	MDR
RVC	VP-16, CsA	1.3	3.8	1.7	WT	MDR, Non-MDR
RDC	DOX, CsA	3.1	21	1.9	WT	MDR, Non-MDR

^a^Drug-resistant cell lines were selected under step-wise increase in cytotoxic drug concentrations in the presence or absence of 2 µmol/L cyclosporine (CsA) or 2 µmol/L PSC-833 (PSC); ^b^fold Resistance to selecting agents, the typical P-glycoprotein (P-gp) substrate vinblastine (VBL), and the weaker P-gp substrate etoposide (VP-16), as determined by comparing the IC50 ratios between drug-selected variants and the parental cell lines (indicated by asterisks); ^c^*ABCB1* cDNAs were reverse-transcribed from mRNAs that contains the full-length *ABCB1* coding sequence and sequenced using the methods described under the Material and Methods section. “Negative” indicates no *ABCB1* mRNA expression; ^d^drug resistance mechanisms classified by ABCB1-mediated multidrug resistance (MDR) and other non-MDR mechanisms; ^e^deletion of the TTC codon (1427-1429) of the *ABCB1* gene, resulting in the absence of the residue phenylalanine 335 (F335del) at the transmembrane region 6 (TM6) of P-gp. CsA: cyclosporine; DOX: doxorubicin; KCVB2 and KPTA5: K562 cells selected with the vinblastine- cyclosporine and paclitaxel-cyclosporine combinations, respectively; KCVB2^i^ and KPTA5^i^: intermediate step cell lines of KCVB2 and KPTA5, respectively; NA: not available; PSC: SDZ PSC 833, the cyclosporin D analog, also known as valspodar; TAX: taxol, also known as paclitaxel; Unstable: unstable drug-resistant phenotypes; VBL: vinblastine; VP-16: etoposide; WT: wild-type

### Cancer specimens from patients

There were 24 cancer specimens from patients used in this study. The Stanford University Committee for the Protection of Human Subjects approved this study, and all patients provided informed written consent (1994). All protocols that involved the collection and use of patents’ samples were approved by the Panel on Medical Human Subjects of Stanford University (2001).

### Drug sensitivity assays

Drug sensitivity or resistance was determined by the MTT [3-(4,5-dimethylthiazol-2-yl)-2,5-diphenyltetrazolium bromide] assay in quadruplicate in 96-well plates, as previously described^[27]^. The resistance factor (Rf) was determined by calculating the ratio of the IC_50_ values (from cytotoxicity curves) between drug-resistant and parental cells.

### Flow cytometric analysis

**Figure 2:**
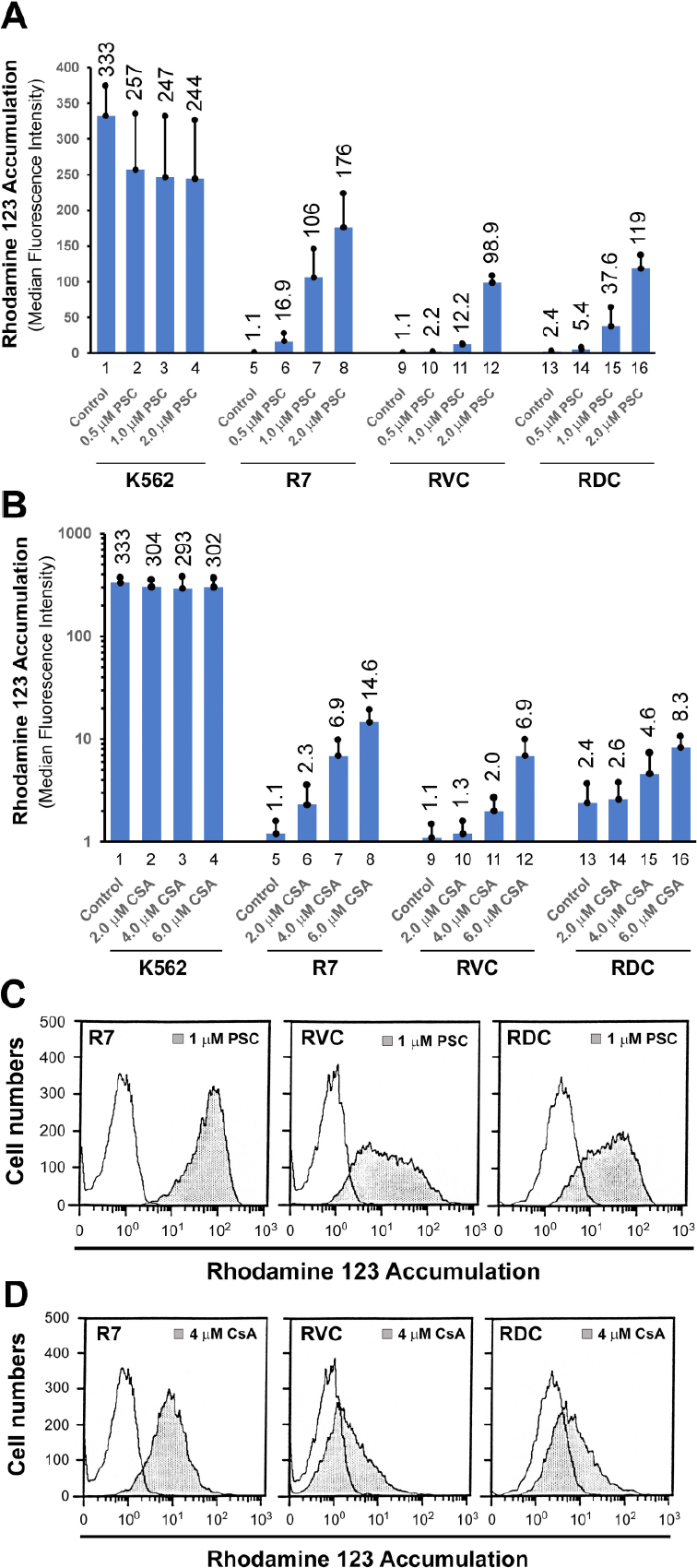
Rhodamine 123 (Rh-123) accumulation assays. A and B: Rh-123 accumulation in the presence or absence of cyclosporine (CsA) and PSC-833 (PSC) in K562, R7, RVC, and RDC cells. The mean values (columns) and standard deviations (bars) were calculated from three independent experiments, with the mean values labeled on the top of the histograms; C and D: Representative histograms of Rh-123 accumulation in R7, RVC and RDC cells in the presence of 1 µmol/L PSC-833 and 4 µmol/L CsA

K562, K562/R7, RVC and RDC cells were grown as suspension cultures, counted, filtered through a nylon filter, and centrifuged at 4 °C. The cell pellets were resuspended in modified Hank’s Balanced Salt Solution (HBSS, Gibco, Life Technologies Inc., MD, USA), which contained 10 mmol/L 4-(2-hydroxyethyl)-l-piperazineethane sulfonic acid and 5% newborn calf serum. Approximately 3 × 10^5^ cells were incubated with CsA and PSC-833 at various indicated concentrations [Figure 2] for 1 h prior to the addition of Rh-123. Rh-123 was added to a final concentration of 0.1 µg/mL, followed by a 45-minute incubation at 37 °C. Cells were centrifuged, and the pellet resuspended in 1 mL of HBSS in the absence or presence of the MDR inhibitors PSC-833 and CsA. Following 20-min of drug efflux at 37 °C, cells were pelleted and resuspended in 200 µL of ice-cold modified HBSS. Retention of Rh-123 was determined by a dual laser flow cytometer (FACS-II; Becton-Dickinson Corp., Mountain View, CA). The median fluorescence intensity was obtained in three independent experiments using the earliest version (1993) of the FlowJo software (Stanford University, CA, USA).

### [^3^H]-labeled drug accumulation

Due to the unavailability of [^3^H]-doxorubicin at the time (around 1993) of experimentation, [^3^H]-daunorubicin was used as the best replacement of [^3^H]-doxorubicin for measuring intracellular doxorubicin accumulation. Intracellular drug accumulation was implemented by adding [^3^H]-daunorubicin (4 µmol/L), [^3^H]-vinblastine (50 nmol/L), and [^3^H]-etoposide (10 mmol/L) to exponentially growing cells. After incubation at 37 °C for 1 h, cells were spun through Nyosil oil to separate them from drug-containing medium. The cell pellets were solubilized in 4% sodium dodecyl sulfate (SDS) solution, incubated at 65 °C for 1 hour, and suspended with the Ecolite Liquid Scintillation Cocktail (MP Biomedicals, Inc., USA). The radioactivity in these samples was measured with a Beckman LS-9000 scintillation counter.

### Reverse transcriptase-PCR and sequencing

The oligonucleotide PCR primers used in this study were synthesized by Ana-Gene Inc. (Palo Alto, CA, USA). These primers, some of which were designed with 5’ *Bam*HI or *Eco*RI restriction sites to facilitate subcloning, were documented previously^[21,27]^. The PCR products were subcloned and sequenced by using the USB Sequenase version 2.0 sequencing kit (Amersham Pharmacia, USA). The results were verified by sequencing the double-stranded cDNAs (obtained from drug-resistant cell lines at various developmental stages). Moreover, the sequencing results were also confirmed by directly sequencing reverse transcriptase-PCR (RT-PCR) products (without subcloning steps) by two independent investigators in our laboratory^[21]^.

### DNA heteroduplex analysis

Isolation of total RNAs and RT-PCR were performed essentially as reported^[21,27]^. Briefly, a 325-bp RT-PCR product was obtained by using a primer set spanning the TM6 region of *ABCB1*. *ABCB1* cDNAs from Dx5 and DxP5 were used as positive and negative controls, respectively, for F335del. The 5-μL post-PCR reaction mixes from tested samples were mixed with 5 μL of either Dx5 (wild-type) or DxP (mutant) RT-PCR products. The mixtures were denatured at 95 °C and then allowed to reanneal by gradually reducing the temperature from 95 °C to 25 °C in 30 min. *ABCB1* PCR products were subjected to electrophoresis on 10%-12% polyacrylamide gels and then stained with ethidium bromide. The PCR products were localized with an ultraviolet transilluminator and photographed for further duplex analysis. A super-shifted (heteroduplex) band is an indication of the existence of the mutant ABCB1 (with F335del).

### RNase protection assay

The RNase protection assay, used to examine *ABCB1* mRNA levels and to map the transcription start sites of the *ABCB1* gene, was performed as previously described^[26,30]^. Briefly, total RNAs were prepared from parental K562, K562/R7, RVC and RDC cells. An antisense RNA probe was produced by SP6 RNA polymerase using the 982-bp genomic fragment (of the *ABCB1* proximal promoter) as the template. Total RNAs were hybridized with [^32^P]-radiolabeled antisense RNA probes (~2 × 10^5^ cpm). The mean densitometric values of each individual band in the blot were determined by using ImageJ software (NIH, Bethesda, MD, USA).

### Exome sequencing analysis of variant allele frequencies

To examine the variant spectrum and allele frequency of *ABCB1* in humans, the UCSC Table Browser Tool (www.genome.ucsc.edu) was used to query and export Genome Aggregation Database Exome Variants (gnomAD Exome v2, released on March 6, 2019; gnomad.broadinstitute.org). We verified that the variants exported were only those falling in exons, hence removing those variants that are positioned before and after each exon. Row data exported for each gene of interest (GOI) was filtered to keep unique variants annotated as having passed all filters (“PASS”) [Supplementary Tables 1-5]. Total number of surviving rows per GOI was tallied with the percentage, representing alternate alleles named as single nucleotide variants (“ALT_SNVs”) and multiple nucleotide variants (“ALT_MNVs”). Allele frequencies (AF) observed greater than 0.50 were reported as 1 - AF.

### Mutational analysis of ABCB1 in human leukemias and other types of cancer

The analysis of the TCGA projects was implemented on June 26, 2020, which was based on the Data Release 24.0 on May 7, 2020 at the National Cancer Institute GDC Data Portal (https://portal.gdc.cancer.gov). At the time of the analysis, TCGA projects had a total of 11,315 cancer cases, 22,872 documented genes, and 3,142,246 mutations. The analysis of simple somatic mutations in the cohort was compared with gene mutations in *TP53* (as control), seven frequently mutated genes (i.e., *NRAS*, *KRAS*, *PTPN11*, *NPM1*, *FLT3*, *DNMT3A* and *IDH2*) in leukemias, and four other ABC transporter genes (i.e., *ABCA1*, *ABCC1*, *CFTR* and *ABCG2*) [Supplementary Table 6].

### Analysis of survival rates of cancer cases with ABCB1 mutations in TCGA

We carried out a survival analysis of cancer cases with *ABCB1* mutations versus those with mutations in *TP53* (as control) and in four other ABC transporter genes (i.e., *ABCA1*, *ABCC1*, *CFTR* and *ABCG2*). The analysis was based on the mortality of the cases and the use of the Kaplan-Meier estimator (https://portal.gdc.cancer.gov). The log-rank test was used to test whether the difference in survival duration between the two groups was statistically significant.

### Statistical analysis

Statistical analyses were performed with the Analysis ToolPak in Microsoft Excel 2019 (Microsoft Corporation, Redmond, WA, USA). The data are presented as mean ± standard deviation (SD). Two sample means were compared using the Student *t*-test. For three and more sample comparisons, we used one-way ANOVA (analysis of variance) (single factor) with a default Alpha value of 0.05. A post hoc *t*-test (i.e., two-sample assuming equal variances) was used for significance testing of pairwise samples. The alpha values were adjusted to 0.0167 and 0.0125 for three- and four-sample comparison, respectively, in this study.

## Results

### Destabilizing the stable multidrug resistant (MDR) phenotypes in K562 cells with cyclosporine

We intended to establish leukemic lines that are resistant to modulation by MDR inhibitors. Hence, we co-selected, in a stepwise manner, non-P-gp-expressing erythroleukemic cell line K562 with tubulin-active drugs paclitaxel (Taxol) and vinblastine in the presence of 2 µmol/L CsA. However, following a one-year period of drug selection, we were unable to derive stable drug-resistant lines [Figure 1A and B]. Typically, the selected cells lost drug resistance to the selecting agents following drug-free growth conditions for 2 to 3 weeks. The drug-resistant phenotypes were stabilized when CsA was replaced with PSC-833 in the selection regimens [Figure 1A and B]. The resulting cell lines, KPTA5 and KCVB2, exhibited non-ABC transporter mechanisms that were associated with altered tubulin expression and polymerization^[41,42]^. Nonetheless, the results from CsA-selection regimens suggested that CsA may play a role in delaying the emergence of stable drug-resistant phenotypes.

### Development of MDR leukemic lines that are insensitive to cyclosporine modulation in K562/R7 cells

To derive stable drug-resistant lines that were insensitive to CsA modulation, we co-selected the P-gp-expressing line K562/R7 (R7) with exposure of the cells to VP-16 and doxorubicin in the presence of 2 µmol/L CsA. The derived lines were named RVC and RDC, respectively [Table 1, Figure 1C]. These two cell lines expressed high levels of *ABCB1* mRNA by RT-PCR and high levels of P-gp like the parental R7 line by Western blotting using the monoclonal antibody C219.

Both RVC and RDC cells were moderately resistant (1.7- and 3.1-fold; two-tailed *t*-test, *P* < 0.05) to their selecting drugs, etoposide and doxorubicin respectively, but highly resistant (21-fold) to the typical P-gp substrate vinblastine in RDC cells [Figure 1D-F]. In contrast to R7 cells, whose resistance to doxorubicin was significantly modulated by CsA (97.6%) [Figure 1D, columns 1 and 2], we observed partial modulation of doxorubicin resistance (58%) in RVC cells under identical experimental conditions [Figure 1D, columns 3 and 4]. In particular, RDC cells exhibited complete resistance to the modulatory effect of CsA (Figure 1D: columns 5 and 6; two-tailed *t*-test, *P* > 0.05).

With respect to the capacity of CsA to modulate vinblastine resistance, we observed a complete reversion of vinblastine resistance (45-fold) to the level of parental K562 cells in R7 cells, but partial reversion (~59% of decline) in RVC cells [Figure 1E, columns 3 and 4] and insensitive to CsA modulation in RDC cells (Figure 1E, columns 5 and 6; two-tailed *t*-test, *P* > 0.05). Concerning the ability of CsA to reverse etoposide resistance, we observed 65% of reversion of the resistance in R7 cells, 51% in RVC, and no modulation in RDC cells (Figure 1F, columns 5 and 6; two-tailed *t*-test, *P* > 0.05). Taken together, these data suggested that the co-selected cell lines RVC and RDC had altered MDR phenotypes that were insensitive to the MDR inhibitor CsA.

### Fluorescent dye Rh-123 accumulation

Rh-123 is a typical P-gp substrate, whose accumulation can be easily detected by flow cytometry. In P-gp-negative K562 cells, Rh-123 accumulation, serving as 100% control, was unexpectedly reduced by the potent P-gp inhibitor PSC-833 [Figure 2A, columns 1-4]. The mechanisms underlying the effect of PSC-833 on decreased Rh-123 accumulation in P-gp-negative cells are unclear. However, PSC-833-mediated Rh-123 reduction in K562 cells was of statistical insignificance as determined in the three independent experiments (ANOVA, *P-*value = 0.46). Clearly, Rh-123 accumulation was insensitive to the effects of various CsA concentrations in K562 cells (Figure 2B, columns 1-4; ANOVA, *P-*value = 0.89).

In P-gp-expressing R7 cells, Rh-123 accumulation was strongly decreased compared with K562 control [Figure 2A, columns 1, 5 to 8]. There was a 96- and 160-fold increase in Rh-123 accumulation in R7 cells treated with 1 µmol/L and 2 µmol/L PSC-833, respectively (Figure 2A, columns 5-8, ANOVA; two-tailed *t*-test, *P*-value < 0.0125). However, the decreased Rh-123 accumulation in both RVC and RDC cell lines was less sensitive to 2 µmol/L PSC-833, displaying 30% and 36% reversion, respectively, relative to K562 control under these same conditions (Figure 2A, columns 1, 12 and 16, ANOVA; two-tailed *t*-test, *P*-value < 0.0167).

With respect to CsA modulation, the decreased Rh-123 accumulation in R7 cells (i.e., 0.33% of K562 control) was only reversed to 0.7%, 2.1% and 4.4% of the K562 control level by 2, 4, and 6 µmol/L CsA, respectively (Figure 2B, columns 5 and 8; ANOVA, two-tailed *t*-test, *P*-value < 0.0125). Rh-123 transport in both RVC and RDC cells was less sensitive to CsA modulation compared with R7 cells under these identical conditions. For example, under 4 µmol/L CsA, a 6.3-fold increase, which might be considered statistically insignificant, in Rh-123 accumulation was found in R7 cells (Figure 2B, columns 5 and 7; ANOVA, two-tailed *t*-test, *P*-value = 0.03 > 0.0125). Moreover, there was only a 1.8-fold increase in Rh-123 signal in RVC cells (Figure 2B, columns 9 and 11, ANOVA, two-tailed *t*-test, *P*-value = 0.15) and 1.9-fold increase in Rh-123 concentration in RDC cells (Figure 2B, columns 13 and 15, ANOVA, two-tailed *t*-test, *P*-value = 0.29).

In general, the decreased Rh-123 accumulation in both RVC and RDC cells was relatively sensitive to the modulatory effect of PSC-833 than that of CsA [Figure 2C and D]. Rh-123 accumulation histograms also revealed the existence of two cell populations in both RVC and RDC lines, as indicated by their differential responses to 1 μmol/L PSC-833 treatment [Figure 2C]. Taken together, the Rh-123 accumulation patterns in both RVC and RDC cells support an altered MDR phenotype that may be associated with defective drug transport mechanisms in these cell lines.

### Anticancer drug accumulation in RVC and RDC lines

**Figure 3:**
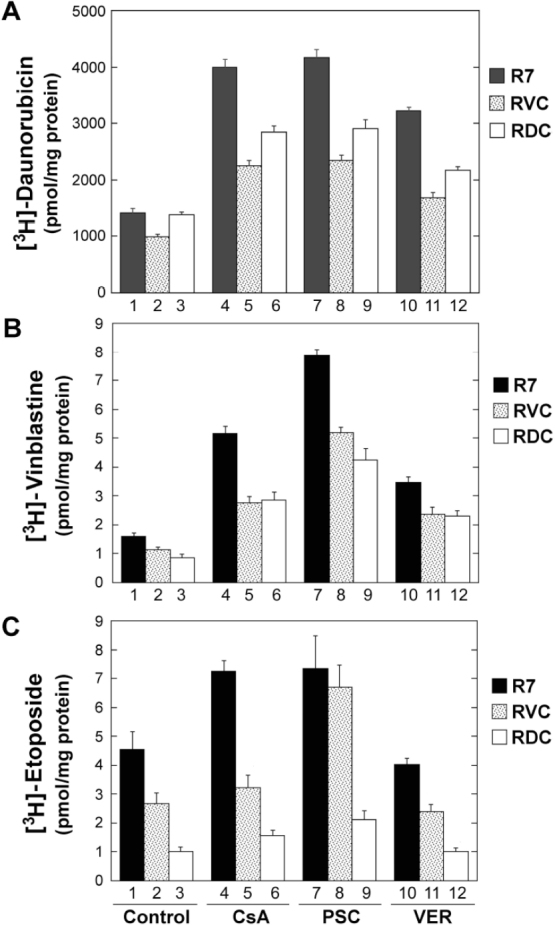
Intracellular cytotoxic drug accumulation. Radiolabeled drugs, including 4 µmol/L [^3^H]-daunorubicin (A), 50 nmol/L [^3^H]-vinblastine (B), and 10 mmol/L [^3^H]-etoposide (C), were incubated with R7, RVC and RDC cells in the presence or absence of cyclosporine (CsA, 4 µmol/L), PSC-833 (PSC, 2 µmol/L), and verapamil (VER, 6 µmol/L). Measurements of the intracellular radiolabeled drugs were performed as described in Materials and Methods. The mean values (columns), also labeled on the top of the histograms, and standard deviations (bars) are derived from quadruplicate determinations. One of two similar experiments is shown

To further define the altered drug transport phenotypes, we evaluated the intracellular accumulation of [^3^H]-labeled drugs. As shown in Figure 3A and Table 2, [^3^H]-daunorubicin accumulation was increased 2.9-, 3.0- and 2.3-fold in R7 cells treated with CsA, PSC-833 and verapamil, respectively. However, [^3^H]-daunorubicin accumulation was relatively insensitive to these MDR inhibitors, only displaying a 2.3-, 2.3- and 1.7-fold increase in RVC cells and a 2.1-, 2.2- and 1.6-fold increase in RDC cells under identical conditions [Figure 3A, Table 2].

**Table 2:** Comparative analysis of drug export in multidrug resistant cell lines

Drugs	R7	RVC	RDC
[^3^H]-Daunorubicin	1*	1*	1*
+ CsA	2.9	2.3	2.1
+ PSC	3.0	2.3	2.2
+ VER	2.3	1.7	1.6
[^3^H]-Vinblastine	1*	1*	1*
+ CsA	3.3	2.5	3.3
+ PSC	4.9	4.7	5.0
+ VER	2.2	2.1	2.7
[^3^H]-Etoposide	1*	1*	1*
+ CsA	1.6	1.2	1.6
+ PSC	1.6	2.5	2.1
+ VER	0.9	0.9	1.0

Fold modulation of [^3^H]-labeled drug accumulation by P-gp inhibitors was determined by comparing the mean values of pmol values of the labeled drugs per milligram protein (as shown in Figure 3) relative to untreated controls (100%, indicated by asterisks). CsA: cyclosporine; PSC: SDZ PSC-833, the cyclosporin D analog, also known as valspodar; VBL: vinblastine; VER: verapamil; VP-16: etoposide

Moreover, [^3^H]-vinblastine accumulation in R7 cells (control) was increased 3.3-, 4.9-, and 2.2-fold in these cells treated with CsA, PSC-833 and verapamil, respectively [Figure 3B, columns 1, 4, 7 and 10; Table 2]. However, there were similar [^3^H]-vinblastine accumulation patterns in both RVC and RDC cells, displaying a 2.5-, 4.7- and 2.1-fold increase in RVC cells (two-tailed *t*-test, *P*-values < 0.05) and a 3.3-, 5.0- and 2.7-fold increase in RDC cells (two-tailed *t*-test, *P*-values < 0.05) under these identical modulation conditions [Figure 3B, Table 2]. Thus, the [^3^H]-vinblastine accumulation patterns suggested the existence of a functional P-gp status in both RVC and RDC cells.

In addition, there was a 1.6-, 1.6- and 0.9-fold increase in [^3^H]-etoposide accumulation in R7 cells treated with CsA, PSC-833 and verapamil, respectively. [^3^H]-etoposide uptake was significantly decreased in both RVC and RDC, exhibiting a 1.7- and 4.5-fold reduction in drug accumulation in RVC and RDC cells, respectively, as compared to R7 cells (Figure 3C, columns 1-3; one-tailed *t*-test, *P-*values < 0.05). Likewise, this deficiency pattern was unable to be reversed to the level of parental R7 cells when RVC and RDC cells were treated with the non-specific P-gp inhibitors CsA and verapamil (Figure 3C, columns 2, 5 and 11; two-tailed *t*-test, *P-*values > 0.05). However, the decreased [^3^H]-etoposide accumulation in RVC cells was significantly reversed by 2 µmol/L PSC-833 (Figure 3C, columns 2 and 8; two-tailed *t*-test *P-*value < 0.05). Clearly, the decreased [^3^H]-etoposide accumulation in RDC cells was relatively insensitive to the modulatory effects of the three MDR inhibitors [Figure 3C, columns 3, 6, 9 and 12] compared to RVC cells. These data indicated that the decreased [^3^H]-etoposide accumulation was likely mediated by the effects of a mutant P-gp or non-P-gp transporters.

Taken together, [^3^H]-labeled drug uptake together with Rh-123 retention revealed a decreased drug accumulation in both RVC and RDC cells, which was resistant to MDR modulators. Thus, these results might be consistent with the evolution of a mutant P-gp, or post-translationally modified P-gp, or non-P-gp drug resistance mechanisms, all of which may render cells resistant to both the selecting drugs (i.e., doxorubicin and etoposide) and the MDR modulator cyclosporine.

### Phe^335^ (F335) deletion in R7, RVC, RDC and human cancers

We initially focused on the identification of potential *ABCB1* exonic mutations or variations in R7, RVC and RDC cells, including a previously identified P-gp mutation (i.e., F335 deletion or F335del) in our laboratory^[21,22]^. F335del in P-gp was identified in a mutant MDR line (DxP), derived from MES-SA/Dx5 cells by stepwise co-selection of the cells with doxorubicin and PSC-833^[21,22]^. P-gp with F335del rendered DxP cells resistant to the reversing effects of MDR inhibitors such as CsA and PSC-833^[21]^. We further examined the frequency of this mutation in R7, RVC and RDC cells, as well as, in 24 *ABCB1*-expressing human cancer samples. These samples included 9 leukemias, 6 lymphomas, and 6 solid malignant tumors. Using mutant *ABCB1* RT-PCR products from DxP cells as the template, we performed RT-PCR and cDNA duplex assays^[21]^. However, the assays failed to detect the 1247-1249 TTC (i.e., the F335 codon) deletion in *ABCB1* cDNAs in all 24 cancer samples, which suggested that F335del might be an uncommon event for human *ABCB1* in drug-selected cancer cell lines and in human cancer patients.

### *ABCB1* sequencing analysis in R7, RVC, RDC and KCVB2 cells

We next manually sequenced the whole coding regions of the *ABCB1* gene in R7, RVC, RDC and KCVB2^i^. KCVB2^i^ is an intermediate line of KCVB2, which had a low level of P-gp expression [Table 1]. We found no mutations in the coding regions of *ABCB1* in R7, RVC, RDC and KCVB2^i^ cells [Table 1]. These data suggested that the *ABCB1* coding regions are stable and resistant to drug-induced mutations in human leukemia cells. Thus, our data ruled out the possibility that the altered MDR phenotypes in both RVC and RDC were due to the contribution of a mutant P-gp.

### Upstream *ABCB1* activation in leukemic MDR cell lines

**Figure 4:**
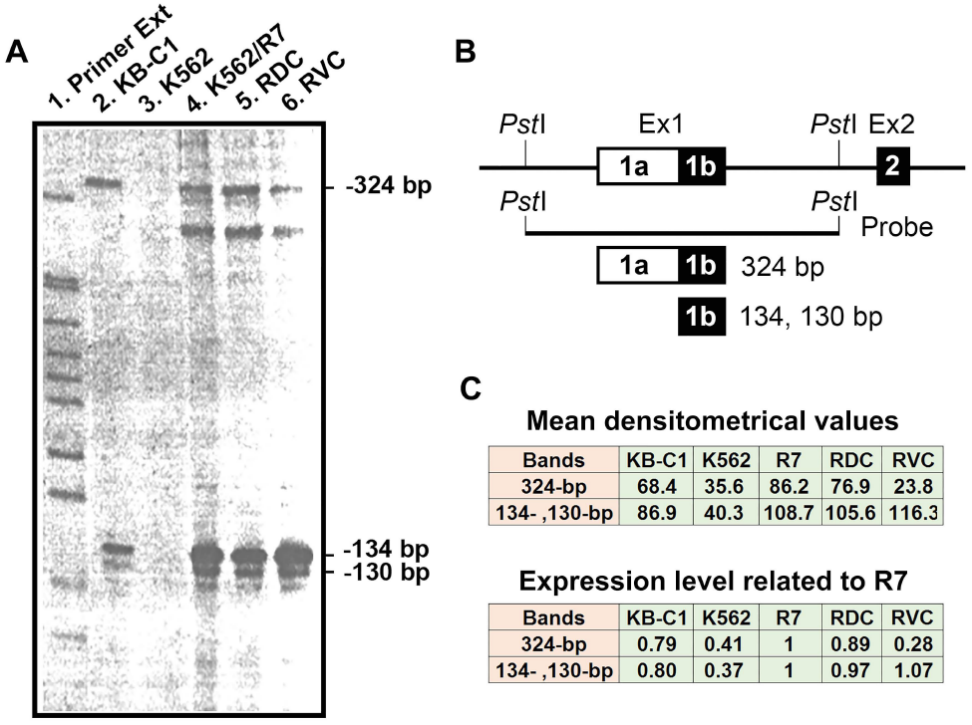
RNase protection analysis of upstream transcriptional start sites. A: total RNAs, extracted from K562 parental cells (*ABCB1* mRNA negative control), KB-C1 cells (*ABCB1* mRNA positive control), and three drug-resistant variants (K562/R7, RVC and RDC), were hybridized with the antisense RNA probe described below in Figure 4B. Primer extension products (lane 1) were used to map the locations of the transcription starting sites (-324-bp, and -130/-134-bp) upstream of the translation starting codon of the *ABCB1* gene; B: structural presentation of the genomic probe (the 982-bp *Pst*I-*Pst*I genomic fragment) of the *ABCB1* proximal promoter used for generating the antisense probe used in the RNase protection assays; C: the densitometric values (i.e., the mean density of the area/band of interest) are determined by using the ImageJ software. *ABCB1* mRNA transcript levels, relative to R7 cells, were determined by calculating the ratios of the mean densitometric values between the cells of interest and R7 cells

To examine the role of *ABCB1* regulation in drug-resistant RVC and RDC cells, we used an RNase protection assay to determine *ABCB1* mRNA expression and to map the transcription start sites of the *ABCB1* gene. As indicated in Figure 4, RNase assay enabled us to identify two protected fragments: one at exon 1b and another one at the upstream regions of exon 1a. Although the exact upstream starting site(s) could not be identified in this assay, the data indicated that *ABCB1* expression in these drug-resistant variants was regulated by two different transcriptional mechanisms. Interestingly, doxorubicin-CsA co-selected RDC cells showed a similar RNase protection pattern to that of parental R7 cells, which suggested that this co-selection regimen might have less impact on *ABCB1* mRNA and protein expression. But etoposide-CsA co-selected RVC cells showed a more than 50% decrease in upstream transcripts concomitantly with an increase in *ABCB1* mRNA expression initiated from exon 1b [Figure 4C]. Thus, RVC data indicated a positive selection of the native *ABCB1* promoter (P1) for the regulation of *ABCB1* and P-gp expression in these cells. The lack of *ABCB1* mRNA mutations without significantly altering *ABCB1* upstream transcription prompted us to extend our initial aim to better understand the intractability of *ABCB1* exons to drug-induced or drug-selected mutations in our cancer models. We chose to compare the variations or mutations among diverse ABC drug transporters as a starting point for unraveling alternative drug resistance mechanisms.

### Genetic variability of *ABCB1* exons

There are 48 ABC transporter genes in the human genome, among which *ABCA1*, *ABCB1*, *ABCC1*, *ABCC7*/*CFTR* and *ABCG2* are well-characterized genes. ABCB1, ABCC1 and ABCG2 are well-studied multidrug transporters *in vitro*. ABCA1 functions as an exporter for intracellular cholesterol and certain phospholipids. CFTR, possibly the only ABC transporter that has gating channel activity (i.e., the opening and closing of an ion channel), functions as a chloride and bicarbonate exporter (www.uniprot.org). We compared the allele numbers and frequencies of exonic variants between *ABCB1* and the above four ABC transporter genes via analyzing exome sequencing data from diverse ethnic groups (gnomAD v2.1, gnomad.broadinstitute.org) [Supplementary Tables 1-5].

**Figure 5:**
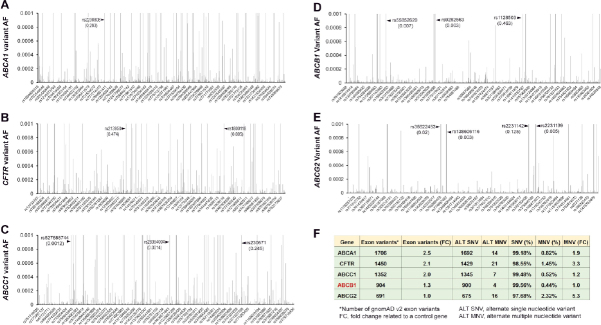
Exonic sequence analysis of *ABCB1* and representative ATP-binding cassette (ABC) transporter genes. A-E: histograms of variant allelic frequencies of exome sequencing data obtained from gnomAD v2.1. Only the allele frequency window between 0 to 0.001 is shown. Some representative alleles with a frequency greater than 0.001 are indicated by arrowheads; F: statistical analysis of validated exon variant numbers of gnomAD v2.1. in five ABC transporter genes with the percentage ratios between SNVs and MNVs indicated

Evidently, *ABCA1*, *CFTR*, *ABCC1* and *ABCB1* displayed a 2.5-, 2.1-, 2.0- and 1.3-fold increase, respectively, in variant allele numbers compared with *ABCG2*, which has the least exon variants [Figure 5A-F, Supplementary Tables 1-5]. These exome sequencing data indicated that both *ABCG2* and *ABCB1* have less exonic variations among the five ABC transporter genes. Moreover, we also examined the percentage of MNVs, which are defined as having two or more variants that coexist on the same haplotype in an individual. We found that *ABCB1* has the least MNV percentage (0.44%), compared with *ABCA1, CFTR, ABCC1 and ABCG2*, which exhibited 1.9-, 3.3-, 1.2 and 5.3-fold increase, respectively, in the percentage of MNVs [Figure 5F]. Interestingly, our previous study reported “TTC” codon deletion (in *ABCB1* cDNA), which is responsible for F335del at TM6 of ABCB1/P-gp^[21]^ and is also a rare MNV variant that is absent from 125,748 human exomes [Supplementary Table 2]. Collectively, these data suggested that *ABCB1* is relatively stable at its exonic regions. MNVs in *ABCB1* may be harmful and thus may be less tolerated than other ABC counterparts by human genomes in the evolutionary process.

### Comparative analysis of the mutational spectrum of *ABCB1* in human AMLs and other types of cancer

**Figure 6:**
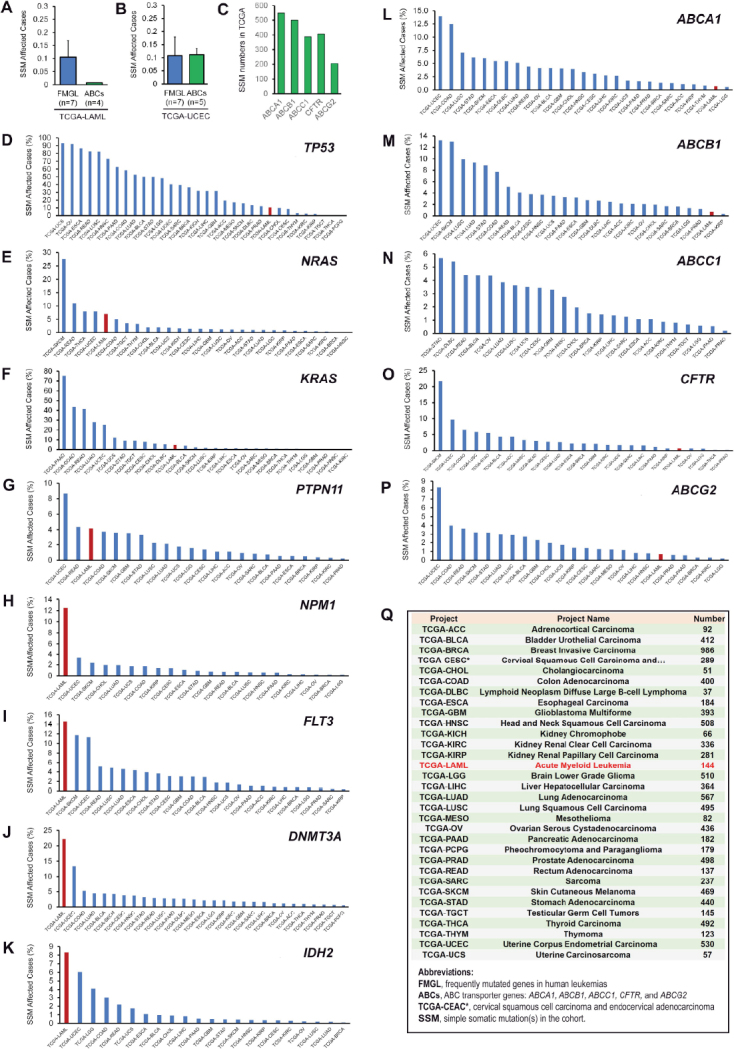
Simple somatic mutational analysis *ABCB1* in human leukemias and other cancer types in the Cancer Genome Atlas (TCGA). A: at the time of analysis, there were 396 cases affected by 550 unique *ABCA1* mutations across 27 projects, 446 cases by 500 *ABCB1* mutations across 26 projects, 324 cases by 389 *ABCC1* mutations across 27 projects, 361 cases by 407 *CFTR* mutations across 25 projects, and 181 cases affected by 206 *ABCG2* mutations across 24 projects; B and C: ABC transporter gene (i.e., *ABCA1*, *ABCB1*, *ABCC1*, *CFTR* and *ABCG2*) mutations were compared with 7 frequently mutated genes (i.e., *NRAS, KRAS, PTPN11, NPM1, FLT3, DNMT3A* and *IDH2*) in leukemas as indicated; D-P: distribution of simple somatic mutations (SSMs) in TCGA projects with the frequency (%) of SSMs in TCGA-LAML (i.e., TCGA project name: acute myeloid leukemia, *n* = 144 cases) indicated by red-colored columns; Q: detailed information and abbreviations with respect to TCGA projects used in Figure 7. SSM affected cases (%) is calculated on the basis of the ratios between the number of cases for SSMs in an individual project affected by each individual gene of interest and the number of cases (as listed in Figure 6Q) tested for SSMs in that project

Currently, TCGA projects at the National Cancer Institute GDC Data Portal (https://portal.gdc.cancer.gov) have annotated and validated a total of 11,315 cancer cases, 22,872 genes, and 3,142,246 mutations. This database offers a great opportunity to define the mutational spectrum of *ABCB1* in human AMLs as well as in other types of cancer [Figure 6]. As shown in Figure 6A, *ABCB1* and three other ABC transporters (*ABCA1*, *CFTR* and *ABCG2*) have a relatively low mutational frequency in AMLs (1/144 or 0.69%) compared to other frequently mutated genes in leukemias (e.g., *NRAS*, *KRAS*, *PTPN11*, *NPM1*, *FLT3*, *DNMT3A* and *IDH2*), which have an average simple somatic mutation (SSM) frequency of 10.1% [Figure 6A]. The 15-fold lower frequency in simple somatic mutations in ABC transporter genes (including *ABCB1*) is of statistical significance in 144 AMLs (unpaired Student *t*-test, *P*-value = 0.013).

In contrast to AMLs, *ABCB1* and other ABC mutational frequencies were greatly elevated across various TCGA projects. For example, *ABCB1* mutational frequency was as much as 13.2% in 530 cases of uterine corpus endometrial carcinoma in the TCGA-UCEC project [Figure 6B, M, Q]. Moreover, the average mutational frequency (i.e., 10.8%) of the frequently mutated leukemia genes (*n* = 7) as described above was almost identical to that (11.1%) of ABC transporter genes (Figure 6B, unpaired Student *t*-test, *P*-value = 0.96). Taken together, these data indicated *ABCB1* exonic stability is cancer cell type-specific (e.g., in both AMLs and renal papillary cell carcinoma) [Figure 6M]. Thus, TCGA data provide a genetic basis that accounts for the genomic stability of the *ABCB1* coding regions in our drug-resistant leukemia models.

### Survival rates of *ABCB1* mutations in TCGA

**Figure 7:**
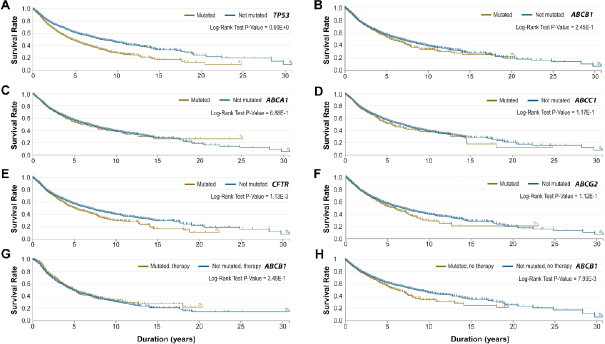
Analysis of survival rates of *ABCB1* mutations in the Cancer Genome Atlas (TCGA). The analysis was compared with cancer cases with gene mutations in *TP53* (as control) and in four other ABC transporter genes (i.e., *ABCA1*, *ABCC1*, *CFTR* and *ABCG2*). The analysis was implemented on June 26, 2020, which was based on the Data Release 24.0 on May 7, 2020 at the National Cancer Institute GDC Data Portal (https://portal.gdc.cancer.gov). At the time of the analysis, the TCGA had a total of 11,315 cancer cases, 22,872 documented genes and 3,142,246 mutations. A-F: the cancer cases for each gene used in this analysis are described as follows: *TP53* mutated (*n* = 4,334) and not mutated (*n* = 6,981), *ABCB1* mutated (*n* = 1003) and not mutated (*n* = 10,312), *ABCA1* mutated (*n* = 846) and not mutated (*n* = 10,469), *ABCC1* mutated (*n* = 788) and not mutated (*n* = 10,527), *CFTR* mutated (*n* = 1,209) and not mutated (*n* = 10,106), and *ABCG2* mutated (*n* = 682) and not mutated (*n* = 10,633); G: pharmaceutical therapy in mutated (*n* = 479) and not mutated (n = 4,198) *ABCB1* cases; H: no therapy in mutated (*n* = 711) and not mutated (*n* = 6,728) *ABCB1* cases. Log-rank test *P*-values are indicated in each plot. The *P*-value for *TP53* in Figure 7A shows 0.00E+0 because the *P*-value is extremely low and goes beyond the precision inherent in the code

We assessed the impact of *ABCB1* mutations on the survival rates in TCGA cancer cases [Figure 7]. The *TP53* tumor suppressor gene, in which its mutations have been well-studied, was used as control in conjunction with the four ABC transporter genes [Figure 7C-F]. Compared with unmutated *TP53* cases (*n* = 6,981), cancer patients with mutated *TP53* (*n* = 4,334) had a significant decrease in cancer survival rate (Figure 7A, log-rank test, *P*-value = 0.00E+0). However, the mutations in *ABCB1*, *ABCA1*, *ABCC1* and *ABCG2* did not affect overall cancer survival rates as determined by log-rank tests (all *P*-values < 0.05). Interestingly, *CFTR* mutations significantly reduced overall cancer survival rates (log-rank test, *P*-value = 1.13E-3), consistent with a possible tumor suppressor function of CFTR in human cancer^[46,47]^.

With regard to the effects of “pharmaceutical” therapy on cancer survival, we found that the overall survival rate from cancer patients with mutated *ABCB1* (*n* = 479) was not statistically different from those with unmutated *ABCB1* control (*n* = 4,198) [Figure 7G]. However, under the “no therapy” condition, the overall cancer survival rate in cancer patients with mutated *ABCB1* (*n* = 711) was indeed significantly decreased when compared with unmutated *ABCB1* control (*n* = 6,278) (Figure 7H, log-rank test, *P*-value = 7.93E-3). These data suggested a possible role of *ABCB1* mutations in impeding cancer survival in certain subsets of cancer patients, likely via gain-of-function mutations that modulate the drug efflux pump.

## Discussion

Concerning the coordinate regulation of MDR and alternative drug resistance mechanisms, there are two outstanding questions that need to be addressed. (1) Do *ABCB1* mutations play a major role in conferring insensitivity to MDR modulation in human leukemias and other types of cancer? (2) How do regulatory mechanisms of *ABCB1* facilitate the development of non-*ABCB1*/P-gp mechanisms? The first question is readily answered for human leukemias on the basis of our findings presented in this study [Figures 1-7]. The lack of induced or selected *ABCB1* mutations in our drug-resistant leukemia models, the genomic stability of *ABCB1* exons, and the low frequency of *ABCB1* mutations in AMLs suggest that mutations in this transporter do not play a significant role in rendering leukemia cells resistant to MDR modulation.

With respect to other cancers, the answer is not clear. *ABCB1* mutations do not appear to contribute to decreased survival rates based on the current TCGA projects [Figure 7B]. This seems to be the case in the subset of patients with “pharmaceutical” therapies [Figure 7G]. Interestingly, in cancer patients without therapy, *ABCB1* mutations indeed has a significant impact on reducing cancer survival rates [Figure 7H], likely reflecting gain-of-function mutations of *ABCB1* in these groups. Noteworthy, the interpretation of *ABCB1* mutations in cancer therapeutics could be influenced by cancer types, therapeutic regimens, and *ABCB1* mutational classes. Unfortunately, some of these factors are not available in the current TCGA database. As more *ABCB1* sequencing data and detailed therapeutic information are included in TCGA, the contribution of *ABCB1* mutations to survival rates of major types of cancer may be assessed in the years to come.

**Figure 8:**
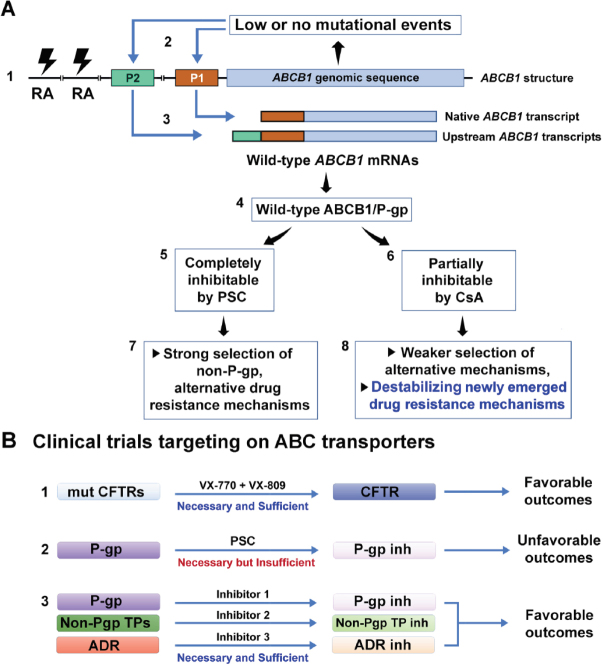
Major pathways that lead to the development of alternative drug resistance mechanisms (ADR) in human cancers. A: the model is based on the evidence from K562/R7, RVC, RDC and other drug-resistant cell lines (described in both Tables 1 and 2). The model depicts that the low to no mutational events of the *ABCB1* coding regions lead to expression or overexpression of wild-type ABCB1/P-gp, whose functional inhibition and partial inhibition encourage the development of stable and unstable ADR mechanisms, respectively; B: therapeutic strategies related to clinical trials that target ABC transporters. CsA: cyclosporine; RA: gene rearrangements; Inh: inhibition; mut: mutant(s); P1: the native *ABCB1* promoter located at exon 1 of the gene; P2: the far upstream *ABCB1* promoter located at exon -1 of the gene; P-gp: P-glycoprotein; PSC: SDZ PSC-833 known as valspodar; TPs: transporters; VX-770: the potentiator drug VX-770 (known as ivacaftor) that targets the CFTR-G551D mutation; VX-809: the corrector drug VX-809 (lumacaftor) that targets the CFTR-F508del mutation

To address the second question, we propose a drug resistance coordination model that links *ABCB1* mutational events to the activation of *ABCB1* and to the induction or selection of alternate drug resistance mechanisms. As illustrated in Figure 8, the low to no mutational events of the *ABCB1* coding regions might lead to expression or overexpression of *ABCB1* by stably transcribing the native *ABCB1* promoter (designated as P1) or the upstream *ABCB1* promoter (P2) [Figures 4A and 8A1], and by selection of potential gene rearrangements at the upstream regions [Figure 8A1]^[26,28-30]^. Here, we speculate that the effect of a mutant *ABCB1* allele might be comparable to that of overexpression of wild-type ABCB1/P-gp. Possibly, the stably overexpressed wild-type ABCB1/P-gp might offer cancer cells a mechanism that compensates the effects of a potential mutant ABCB1/P-gp (e.g., F335del) on drug-binding and cytotoxic insults as previously described^[20-22,48]^. Thus, the expressed wild-type P-gp is partially inhibitable by weaker P-gp inhibitors (e.g., CsA) and completely inhibitable by potent and specific P-gp inhibitors (e.g., PSC-833). Such differential inhibition of P-gp function would conceivably lead to two different trajectories for the development of non-P-gp-mediated mechanisms [Figure 8A4-8].

In the case of CsA, it partially inhibits the function of P-gp as well as other multidrug transporters such as ABCG2 and ABCC1^[49]^. The benefit of such a broad and partial inhibition of diverse multidrug transporters would make cancer cells moderately resistant to anticancer drugs. The CsA-mediated inhibition might also destabilize the newly emerged drug resistance mechanisms [Figures 1 and 8A, Table 1], which may be partially associated with the capacity of CsA to induce apoptosis in some human cancer cells^[50]^. However, the disadvantage of such inhibition would be that it could not eradicate MDR cells. Overall, CsA contributes to deferring stable drug-resistant phenotypes in RVC, RDC and KCVB2 cell lines [Table 1, Figure 1A and B], which at least partially explains the benefits of CsA in reversing clinical MDR in P-gp-expressing leukemia patients^[9]^.

In the case of PSC-833, which represents a potent and more specific P-gp inhibitor, it greatly inhibits the function of P-gp as well as a few multidrug transporters such as ABCG2 and ABCC1^[1,3]^. A strong inhibition of a narrow range of multidrug transporters would likely sensitize cancer cells to anticancer drugs that are typical P-gp substrates. However, such inhibitory effects would also permit the selection or induction of non-ABC transporter-mediated drug resistance. This might explain the selection of relatively stable drug-resistant phenotypes in KCVB2, KPTA5 and MES-SA/DxP cell lines using PSC-833 rather than CsA [Figure 8A]^[21,41,42]^. The coexistence of alternative drug resistance mechanisms is likely to explain the unfavorable outcomes identified in the clinical trials that used PSC-833 and other potent P-gp inhibitors as modulators of MDR.

Whether the inhibition of *ABCB1*/P-gp is a rational therapeutic approach to circumventing clinical MDR seems to be unfavorable because of no supportive clinical data. However, we may address this complicated question by asking a simple one: is targeting an ABC transporter a rational strategy to treat ABC transporter-related diseases? The answer to this question is yes. As exemplified by CFTR, an ABC transporter that functions as a chloride channel, its dominant mutations are now widely used as molecular targets for therapy of patients with cystic fibrosis. The recent proof-of-concept experiments using stem-cell organoids from patients with cystic fibrosis confirmed that targeting CFTR with small molecules is both rational and effective^[51,52]^.

However, in contrast to patients with cystic fibrosis, which is principally associated with only the ABC transporter CFTR, patients with refractory cancer usually express several ABC transporter-dependent and -independent mechanisms. Thus, targeting ABC multidrug transporters for treating patients with MDR can be complicated by the redundancy of multidrug transporters and alternative drug resistance mechanisms. Thus, it is conceivable that P-gp inhibition is still necessary, but insufficient, for the reversal of clinical MDR [Figure 8B2]. A combined inhibitory regimen that targets P-gp, non-P-gp transporters, and other drug resistance mechanisms might be both necessary and sufficient to reverse clinical MDR [Figure 8B3]. Alternatively, MDR may be circumvented clinically by using anticancer agents that are not transport substrates for P-gp and other transporters.

Currently, evaluating cancer drug response in animal models is difficult as human cancer patients often have considerable genetic heterogeneity, with various non-P-gp genomic mutations and epigenomic states. Cancer stem cell-derived 3D organoids may faithfully retain genetic epigenetic signatures of original cancer tissues^[53,54]^. Therefore, it is possible that cancer organoids from drug-resistant patient groups might be classified, assayed and used for predicting drug responders in patients with MDR *in vitro*.

Clinical drug resistance is a complicated genetic phenotype, which is attributable to the predominant multidrug transporter ABCB1/P-gp, miscellaneous non-P-gp transporters, and other drug resistance mechanisms. Our study in leukemia models revealed that *ABCB1* coding regions are stable even under stringent drug selection, thus suggesting that a mutant P-gp may not be a major factor responsible for the unsuccessful modulation of clinical MDR. *ABCB1* mutational data in AMLs in TCGA seem to support our findings and suggestions. In addition, partial inhibition of P-gp and other ABC drug transporters by CsA favors induction of unstable drug-resistant phenotypes, which might delay the onset of drug-resistant cells under certain circumstances and benefit some cancer patients. Finally, we propose the drug resistance coordination model that elucidates the rationale for P-gp inhibition as a treatment approach for cancer patients, highlighting that co-inhibition of P-gp and non-P-gp-mediated drug resistance may be required for overcoming MDR in refractory cancer patients.

## Declarations

### Availability of data and materials

The datasets supporting the conclusions of this article are included within the article and Supplementary Tables.

### Copyright

© The Author(s) 2022.
